# Sleep Disorders in Rodent Models of Parkinson’s Disease

**DOI:** 10.3389/fphar.2019.01414

**Published:** 2019-11-27

**Authors:** Daniel de Castro Medeiros, Cleiton Lopes Aguiar, Márcio Flávio Dutra Moraes, Gilberto Fisone

**Affiliations:** ^1^Department of Neuroscience, Karolinska Institutet, Stockholm, Sweden; ^2^Núcleo de Neurociências, Departamento de Fisiologia e Biofísica, Instituto de Ciências Biológicas, Universidade Federal de Minas Gerais, Belo Horizonte, Brazil

**Keywords:** sleep, rapid eye movement sleep, slow wave sleep, rat, mouse, disease models, 6-hydroxydopamine, 1-methyl-4-phenyl-1,2,3,6-tetrahydropyridine

## Abstract

Sleep disorders are frequently diagnosed in Parkinson’s disease and manifested in the prodromal and advanced stages of the disease. These conditions, which in some cases affect more than 50% of Parkinson’s disease (PD) patients, include hypersomnia, often manifested as excessive daytime sleepiness, insomnia, characterized by delayed initiation and fragmentation of sleep at night, and disruption of rapid eye movement (REM) sleep, resulting in loss of atonia and dream enactment. Standard dopamine replacement therapies for the treatment of motor symptoms are generally inadequate to combat sleep abnormalities, which seriously affect the quality of life of PD patients. Rodent models still represent a major tool for the study of many aspects of PD. They have been primarily designed to eliminate midbrain dopamine neurons and elicit motor impairment, which are the traditional pathological features of PD. However, rodent models are increasingly employed to investigate non-motor symptoms, which are often caused by degenerative processes affecting multiple monoaminergic and peptidergic structures. This review describes how neurotoxic and genetic manipulations of rats and mice have been utilized to reproduce some of the major sleep disturbances associated with PD and to what extent these abnormalities can be linked to nondopaminergic dysfunction, affecting for instance noradrenaline, serotonin, and orexin transmission. Strengths and limitations are discussed, as well as the consistency of results obtained so far, and the need for models that better reproduce the multisystemic neurodegenerative nature of PD, thereby allowing to replicate the complex etiology of sleep-related disorders.

## Introduction

Since their first description two centuries ago, the symptoms of Parkinson’s disease (PD) have been regularly re-assessed to include a large number of nonmotor conditions affecting both the peripheral and the central nervous system (Chaudhuri and Odin, 2010; Lang, 2011). This has occurred in parallel with the recognition of the complexity of the neurodegenerative processes at the basis of PD, whose definition extends beyond that of a simple dopaminergic disorder. Indeed, accurate analyses of the spread of α-synuclein pathology from the peripheral nervous system, lower brainstem, and olfactory bulb, to the diencephalon, basal forebrain, and neocortex, show that PD affects not only dopamine, but also noradrenaline, serotonin, and acetylcholine containing structures (Braak et al., 2003).

This diversified pathology has been correlated to the gradual appearance of non-motor symptoms. For instance, gastrointestinal, bladder, and olfactory dysfunctions, which are common preclinical features of PD, have been linked to the initial stage of the disease, affecting the autonomic nervous system and olfactory nuclei. Other conditions, including sleep disturbances and affective disorders appear in conjunction with the caudal-rostral spread of α-synuclein deposition to the locus ceruleus, raphe, and lateral tegmental nuclei. Additional non-motor symptoms include cognitive dysfunction, often developing into dementia during the late stages of PD (Chaudhuri and Odin, 2010; Lang, 2011).

Sleep disorders are among the most frequent symptoms observed in PD patients, with a prevalence of 60–70% (De Cock et al., 2008; Barone et al., 2009; Neikrug et al., 2013). They often appear in the early stages of the disease and are considered as markers of prodromal parkinsonism (Schenck et al., 1996; Postuma et al., 2010; Lysen et al., 2019). Sleep-related symptoms in PD consist of a broad spectrum of conditions, which include hypersomnia, the difficulty in initiating and maintaining sleep and disturbances of rapid eye movement (REM) sleep. These disorders progressively worsen in the course of PD and represent a major factor affecting the patient’s quality of life (Maetzler et al., 2009; Neikrug et al., 2013). They are generally refractory to, or exacerbated, by standard anti-parkinsonian medications. In this context, it should be mentioned that impaired synaptic downscaling associated with sleep disruption may promote the emergence of motor complications caused by standard pharmacological therapy (Galati et al., 2015), and that this idea is supported by clinical studies indicating a correlation between disrupted sleep and l-3,4-dihydroxyphenylalanine (L-DOPA)-induced dyskinesia (Mao et al., 2018).

Several studies show that surgical treatment, specifically deep brain stimulation targeting the subthalamic nucleus, ameliorates sleep quality in PD (Arnulf et al., 2000; Antonini et al., 2004; Cicolin et al., 2004; Hjort et al., 2004; Kharkar et al., 2018). This could be explained by the ability of this intervention to counteract motor disturbances (Arnulf et al., 2000), but additional effects possibly exerted on sleep structures cannot be excluded (Kharkar et al., 2018). In spite of these efforts, the progressive deterioration of sleep observed in a large proportion of patients still represents a major problem in the management of PD.

In this review, we describe and discuss current attempts to study PD-related sleep disorders in rodents, which represent the most common experimental animals used to model motor and non-motor symptoms of PD.

### Sleep Physiology

Sleep is a natural and reversible state, defined by lack of mobility (or slight mobility) and relative unresponsiveness to internal and external stimuli (Carskadon and Dement, 2017), closely associated with the patient’s general health condition and necessary for optimal cognitive functions (Scammell et al., 2017). In general, a typical sleep period encompasses recurrent cycle successions of two states with distinct physiological characteristics, as assessed by electroencephalography (EEG), and electromyography (EMG): non-REM (NREM) and REM sleep (Chokroverty, 2009). During NREM sleep, the EMG indicates a decrease of muscle activity and the EEG shows a predominance of slow oscillations (0.5–4 Hz). For this reason, NREM sleep is also referred to as slow-wave sleep (SWS). In contrast, the REM phase presents a desynchronized EEG pattern (similar to wakefulness) along with a drastic reduction of muscle tonus (Lee and Dan, 2012).

The sleep-wake brain states and muscle tonus are mainly regulated by the posterior hypothalamus and several nuclei in the brainstem, collectively named ascending activating (or arousal) system (AAS). This system projects to the thalamus, basal forebrain, neocortex, and to the spinal cord (Fuller et al., 2016) and is essential for arousal and wakefulness (Moruzzi and Magoun, 1949). The firing rate of the neural components in the AAS, which consist mainly of monoaminergic and cholinergic neurons, changes along NREM-REM sleep cycles (Jones, 2003). The sleep monoaminergic nuclei complex includes the noradrenergic locus coeruleus (LC) (Carter et al., 2010), the serotonergic dorsal and median raphe nuclei (Ito et al., 2013), the dopaminergic ventral periaqueductal gray matter (vPAG) (Lu et al., 2006), and the histaminergic tuberomammillary neurons (TMN) (Yu et al., 2014). These nuclei present high firing rates during the wake period, lower firing during NREM sleep and are almost silent throughout the REM state. Conversely, the cholinergic neurons, which are clustered in the pedunculopontine (PPT) and lateral dorsal tegmental nuclei, fire rapidly during wake and REM periods but slowly during NREM sleep (Boucetta et al., 2014).

The AAS receives inhibitory inputs from galanin and gamma-aminobutyric acid-ergic (GABAergic) neurons located in the ventrolateral preoptic area (VLPO) and median preoptic nucleus (Suntsova et al., 2007). These hypothalamic neurons present a faster firing rate during sleep and effectively shut down the AAS wake-promoting cells (Sherin et al., 1996; Lu et al., 2002). On the other hand, AAS afferents from the LC and dorsal raphe connect to VLPO neurons that are inhibited by norepinephrine and serotonin (Chou et al., 2002). This reciprocal inhibitory control acts as a feedback loop (neural flip-flop circuit), leading to the alternation and stability of sleep-wake competing states (Saper et al., 2001; Saper et al., 2005). In addition, the AAS includes afferent connections from GABAergic neurons in the substantia nigra pars reticulata and from orexins (orexin-A and orexin-B; also known as hypocretin-1 and 2) containing neurons in the lateral hypothalamus (LH) (de Lecea et al., 1998; Sakurai et al., 1998). While the former induces NREM-REM states *via* inhibition of dorsal raphe and LC (Liu and Dan, 2019), the later promotes wake and suppresses sleep (particularly REM sleep) (Scammell et al., 2017) by activating LC and TMN (Peyron et al., 1998).

REM sleep is also controlled by means of a mutual inhibition between neuronal circuits located in the mesopontine tegmentum. The sub-laterodorsal nucleus and precoeruleus region comprise the REM-promoting (REM-on) structures. Glutamatergic neurons projecting from these regions regulate the activity of the basal forebrain and medulla, thereby promoting cortical high-frequency paradoxical oscillations and muscle atonia typical of REM sleep. The REM-off nuclei, i.e., vPAG and lateral pontine tegmentum, provide a REM flip-flop switch arrangement *via* GABAergic inhibition of REM-on nuclei (Peever and Fuller, 2017).

Hypothalamic and brainstem neurotransmitter systems modulate the REM-switch structures and support the sleep cycles. LC noradrenergic and dorsal raphe serotonergic neurons suppress REM sleep by exciting REM-off and inhibiting REM-on areas, whereas lateral dorsal tegmental and PPT cholinergic neurons promote REM by opposite actions on the same REM-on/off populations. Additionally, orexin neurons excite REM-off structures and support sleep-wake stabilization, whereas the VLPO promotes the entry into REM sleep by inhibiting the same targets (Lu et al., 2006; Peever and Fuller, 2017).

Transitions between sleep and wake are thought to be regulated by two main processes—the homeostatic process (process S) and the circadian pacemaker (process C) (Borbély et al., 2016). Prolonged periods of wakefulness are followed by a corrective higher amount of sleep, referred to as sleep rebound. This homeostatic response is mediated by substances (somnogens) that accumulate during the wake periods and dissipate during sleep. One of the best-known somnogens is adenosine, a paracrine mediator produced by the degradation of ATP (Porkka-Heiskanen et al., 1997). Higher extracellular levels of adenosine promote sleep-state by inhibiting the AAS *via* adenosine A1 receptor (Strecker et al., 2000) and stimulating VLPO *via* A2 receptors (Scammell et al., 2001). The circadian pacemaker opposes the homeostatic process during the active period of the sleep-wake cycle, *via* the suprachiasmatic nucleus, which promotes wakefulness *via* excitation of LH orexin neurons and inhibition of VLPO neurons (Saper et al., 2005).

### Sleep Disturbances in Parkinson’s Disease

The progression of PD affects multiple neurotransmitter pathways that extend beyond dopaminergic degeneration in the substantia nigra pars compacta (SNc) (Braak et al., 2004; Surmeier et al., 2017), often comprising structures related to the sleep-wake cycle (French and Muthusamy, 2016). In fact, most PD patients present neuronal cell loss and Lewy bodies in the noradrenergic neurons of the locus coeruleus (Zarow et al., 2003), serotonergic and dopaminergic neurons in medial and dorsal raphe and vPAG (Halliday et al., 1990), as well as cholinergic, histaminergic, and orexinergic neurons in the pedunculopontine nuclei (PPN), TMN, and LH (Zweig et al., 1989; Fronczek et al., 2008; Shan et al., 2012; French and Muthusamy, 2018). Disruptions of these structures and connected circuits are likely to play an important role in sleep disturbances, such as insomnia, excessive daytime sleepiness (EDS), and REM sleep behavior disorder (RBD). In addition to these conditions, sleep in PD is influenced by motor abnormalities, such as restless legs syndrome, which can seriously compromise nocturnal sleep, and breathing disorders, leading to sleep apnea (Mery et al., 2017; Ferini-Strambi et al., 2018). Altogether, these disturbances seriously contribute to fragmented sleep-wake behavior observed in PD.

Insomnia is one of the most common sleep disorders in PD, affecting up to 60% of patients (Gjerstad et al., 2007). It is defined by a repeated difficulty in sleep initiation, duration, consolidation, or quality that occurs despite adequate time or opportunity for sleep (Thorpy, 2009). PD-related insomnia presents a multifactorial etiology and is often manifested as comorbidity rather than a single sleep problem (Ylikoski et al., 2015). The role played in PD-related insomnia by lesions affecting brain structures directly involved in arousal and sleep-wake behavior should not be overlooked. However, a number of motor conditions intrinsic to the disease process or induced by dopamine replacement therapy, such as tremor, restless legs syndrome, nighttime cramps, dystonia, and dyskinesia, play an essential role in the pathophysiology of PD-related insomnia (Arnulf et al., 2000). Similarly, non-motor symptoms including autonomic dysfunction and psychiatric symptoms have also been associated with both insomnia and hypersomnia (Kurtis et al., 2013). Drug-disease interaction is also associated with insomnia in PD patients. For example, D1 and D2 receptors activation by higher doses of dopaminergic medication at bedtime are correlated with poor sleep quality (Chahine et al., 2013).

EDS is diagnosed in 15–50% of PD patients (Ondo et al., 2001) and is characterized by a disabling urge to nod or fall asleep during different daily-life circumstances, with a severe negative impact on the overall quality of life (Arnulf, 2005). The damage in the AAS nuclei is an essential factor in PD related EDS (Arnulf and Leu-Semenescu, 2009), particularly the degeneration of hypothalamic orexin cells (Fronczek et al., 2008), whose activity is crucial for vigilance maintenance (Sakurai, 2007; Ono and Yamanaka, 2017) and for the suppression of pathological intrusions of REM sleep-related events during wakefulness (narcolepsy) (Hara et al., 2001). Additionally, EDS (somnolence) has direct correlation with the dosage of daytime dopamine agonist treatment (Avorn et al., 2005).

RBD is defined by the loss of normal REM atonia with prominent out-of-dreams muscle activation (Schenck et al., 2002), which may result in severe injuries to patients and their family. RBD affects approximately 30% of patients with PD (Barone et al., 2009) and is associated with loss of volume and neuronal degeneration in brainstem REM-on areas (Boeve et al., 2007; Garcia-Lorenzo et al., 2013; Boucetta et al., 2016), which may be caused by the pathological accumulation of α-synuclein (Braak et al., 2004). Additionally, REM-sleep stability may be compromised by multiple degeneration of serotonergic, noradrenergic, cholinergic, and orexinergic nuclei that support and modulate the REM-switching system (Boeve et al., 2007; Fronczek et al., 2007; Garcia-Lorenzo et al., 2013; Boucetta et al., 2016; French and Muthusamy, 2018).

### Rodent Models of Parkinson’s Disease for the Study of Non-Motor Symptoms

The multiple neurodegenerative processes potentially involved in non-motor symptoms contrast with the relatively well-established cause at the basis of the cardinal motor symptoms of PD, i.e., the progressive degeneration of dopamine neurons in the SNc, projecting to the dorsal striatum (DS). Because of the traditional view of PD as a motor disorder, the development of experimental models has been centered on the elimination of the dopamine nigrostriatal pathway. This is generally achieved by local or systemic administration of neurotoxins (Tieu, 2011) or by genetic manipulations (Dawson et al., 2010).

#### Neurotoxin Models of Parkinson’s Disease

Neurotoxin models of PD are commonly based on the use of 6-hydroxydopamine (6-OHDA) (Simola et al., 2007) or 1-methyl-4-phenyl-1,2,3,6-tetrahydropyridine (MPTP) (Meredith and Rademacher, 2011). These compounds act by inhibiting mitochondrial function and generating reactive oxygen species, ultimately leading to oxidative stress and cell death (Simola et al., 2007; Meredith and Rademacher, 2011). In the mouse, repeated systemic administration of MPTP leads to a substantial loss of dopamine neurons and fibers. Depending on dose and regimen, MPTP has been reported to produce from 20 to 70% neuronal loss in the SNc and from 50 to 90% decrease in dopamine levels in the DS (Jackson-Lewis and Przedborski, 2007; Meredith and Rademacher, 2011).

6-OHDA is typically administered *via* stereotaxic injection in the medial forebrain bundle (MFB), dopaminergic midbrain nuclei (e.g., SNc) or directly into the DS of rats and mice. The injection in the MFB produces a nearly complete loss of nigrostriatal dopamine, mimicking end-stage PD (Yuan et al., 2005). This approach is normally utilized in combination with a unilateral lesion since a total bilateral elimination of dopamine would result in high mortality. Bilateral lesions are most frequently performed by injecting 6-OHDA in the DS, which leads to a partial loss of dopamine neurons in the SNc and a reduction of striatal dopamine varying between 45 and 75% (Tadaiesky et al., 2008; Branchi et al., 2010; Bonito-Oliva et al., 2013). This latter approach is regarded as a model of early stage PD (Yuan et al., 2005).

Because of its similarity to endogenous catecholamines, 6-OHDA produces a combined degeneration of dopamine and noradrenaline neurons. This has been described in initial studies in rats (Breese and Traylor, 1971) and observed also in the mouse model of partial striatal lesion (Bonito-Oliva et al., 2014). In addition, both 6-OHDA and MPTP have been shown to decrease serotonin levels in the hippocampus (Reader and Gauthier, 1984; Santiago et al., 2010). The ability of these toxins to affect multiple neurotransmitter systems altered in PD, is especially important for the study of non-motor symptoms.

##### Sleep Disturbances in the 6-Hydroxydopamine Model of Parkinson’s Disease

Rats with a unilateral 6-OHDA lesion of the MFB show decreased sleep time during the light-on, inactive phase of the 24-h light-dark cycle, and increased total wake time assessed during a 24-h recording (Vo et al., 2014). When compared to naïve animals, these animals also show increased muscle activity during REM sleep, which is indicative of RBD-like abnormalities (Vo et al., 2014). Sleep analysis has recently been performed in rats injected unilaterally with 6-OHDA in the SNc, which results in a substantial loss of dopamine neurons (Ciric et al., 2019). This intervention leads to increased number and mean duration of wake episodes assessed by cortical and hippocampal EEG during 6 h within the inactive, light-on period (Ciric et al., 2019). Increased wake time, particularly evident during the 12-h dark period, has also been observed in rats with a selective 6-OHDA lesion of the SNc (Qiu et al., 2016). Conversely, cortical EEG recording in rats injected with 6-OHDA in the lateral ventricle showed decreased wakefulness and augmented SWS (Monti et al., 1999). This difference is likely explained by the more generalized effect on multiple components of the catecholamine system exerted by intracerebroventricular (i.c.v.) administration of 6-OHDA, in comparison to selective stereotactic injections in MFB or SNc (Vo et al., 2014; Ciric et al., 2019).

Sakata et al. investigated the effect of bilateral 6-OHDA lesion of the ventral tegmental area (VTA) on sleep-wake cycles (Sakata et al., 2002). They found reduced REM sleep during the light-on period, in comparison with control rats. The 6-OHDA-lesion rats also displayed reduced wake duration and spontaneous activity accompanied by a significant increase of both REM and SWS (NREM) during the dark phase (Sakata et al., 2002). These findings are in line with some of the disturbances observed in PD patients, who are affected by insomnia at night and daytime sleepiness.

Further studies in rats showed that injection of 6-OHDA in the DS or MFB decreases the number of orexin neurons in the LH (Cui et al., 2010; Oliveira et al., 2018), thereby reproducing a similar damage described in PD (Maeda et al., 2006; Thannickal et al., 2007). This loss was paralleled by reduced baseline respiratory frequency during sleep in the light-on period (Oliveira et al., 2018), a condition reminiscent of breathing disturbances and sleep apnea observed in PD patients (Mery et al., 2017; Videnovic, 2017). The same 6-OHDA lesion did not affect the sleep-wake cycle (Oliveira et al., 2018). This contrasts with previous work in which LH orexinergic neurons were preferentially targeted using a neurotoxin acting through the ribosome-inactivating protein saporin (hypocretin2-saporin) (Gerashchenko et al., 2001). In this case, the authors found increased NREM and REM sleep during the dark, active phase, and sleep fragmentation leading to reduced number of REM sleep episodes during the light-on phase (Gerashchenko et al., 2001). Similar changes of REM and NREM sleep in the light-off period, were observed using a cycad toxin-based PD model (see below), which leads to a significant reduction in orexin neurons (McDowell et al., 2010). It should be noted however that in the 6-OHDA study EEG recordings were performed during the last 3 h of the light period and the first 3 h of the dark period (Oliveira et al., 2018), instead of the more appropriate 24-h continuous recordings, as in the previous studies using saporin or cycad (Gerashchenko et al., 2001; McDowell et al., 2010).

Urethane anesthesia is characterized by alternations of oscillatory states which resemble REM-NREM cycling during natural sleep. Despite the obvious limitation of this approach, a number of studies have performed electrophysiological recordings in rodents under urethane to test mechanistic and functional hypothesis related to sleep-like states (Clement et al., 2008; Gonzalez-Rueda et al., 2018; Hauer et al., 2019). Resting-state functional magnetic resonance has been recently employed to examine modifications of functional connectivity in urethane-anesthetized rats with a unilateral partial striatal 6-OHDA-lesion (Zhurakovskaya et al., 2019). In this study, REM- and NREM-like sleep states were identified based on their association with high and low breathing rates, respectively (Pagliardini et al., 2012; Zhurakovskaya et al., 2019). Rats with a 6-OHDA lesion showed diminished intra-cortical, cortico-hippocampal, and striato-cortical functional connectivity, in comparison to naïve, or sham-lesion rats. Notably, these abnormalities were observed only during REM-like sleep (i.e., during states associated with high breathing rate), and may point toward changes in muscle tone suggestive of RBD (Zhurakovskaya et al., 2019). However, it remains to be established whether these alterations of connectivity can also be observed during sleep.

##### Sleep Disturbances in the 1-Methyl-4-Phenyl-1,2,3,6-Tetrahydropyridine Model of Parkinson’s Disease

Sleep disruptions have also been observed in the MPTP model of PD. However, these alterations are transitory, which could be ascribed to limited or reversible neuronal loss, as well as to parallel compensatory effects (Johannessen et al., 1985; Saitoh et al., 1987; Bezard et al., 2000). Mice treated with five daily injections of MPTP (25 mg/kg) showed increased REM sleep during the 24-h light/dark cycle, in comparison to control. However, this effect was observed only 20 days after toxin injection and disappeared 40 days post MPTP (Monaca et al., 2004; Laloux et al., 2008). Interestingly, the reduction of dopamine neurons produced by MPTP, which was restricted to the SNc and amounted to approximately 30%, remained constant for up to 60 days, suggesting the existence of mechanisms correcting for the neuronal loss produced by the toxin (Laloux et al., 2008). In contrast to the aforementioned work, EEG analysis performed in mice 14 days after a single injection of MPTP (40 mg/kg), reported increased wake, and decreased NREM sleep during the light-off period, but no change in REM sleep. In this study, however, MPTP produced a large reduction of dopamine neurons in both SNc (78%) and VTA (54%) (Revishchin et al., 2016).

A study in the rat, examined the effect on sleep patterns of local injection of MPTP in the SNc, leading to a temporary (24 h) 50% decrease of tyrosine hydroxylase (a marker of dopamine neurons) (Lima et al., 2007). The authors observed a decrease in the latency to SWS onset during both the light-on and light-off periods, which lasted for up to 5 days following MPTP administration. This change was accompanied by increased sleep efficiency and may represent a surrogate marker of daytime sleepiness in PD. The same study also reported a significant increase in the latency to REM sleep in the MPTP rats compared to control animals. However, this disturbance was transient and disappeared 3 days after the administration of MPTP (Lima et al., 2007).

##### Sleep Disturbances in Other Toxin-Based Models of Parkinson’s Disease

Chronic administration of rotenone, a natural compound commonly used as a pesticide, leads to a significant degeneration of SNc dopamine neurons accompanied by α-synuclein inclusions (Betarbet et al., 2000; Alam and Schmidt, 2002). Working with a rotenone-based rat model of PD, Yi et al. reported increased SWS and REM sleep during the light-off active period and decreased SWS during the light-on period (Yi et al., 2007). These modifications, which are suggestive of PD-related daytime sleepiness and nighttime insomnia, were largely refractory to L-DOPA. Notably, the effect of rotenone was accompanied by increased hypothalamic levels of interleukin-1β, a cytokine previously shown to promote REM and NREM sleep (Krueger, 2008). In line with this observation, i.c.v. administration of the endogenous interleukin-1 receptor antagonist (IL-1RA) counteracted the changes in SWS produced by rotenone during both the active and inactive 12-h periods (Yi et al., 2007). A word of caution should be spent concerning the solution (1:1 DMSO/PEG) commonly used to dissolve rotenone. In one study, this vehicle precluded a conclusive interpretation of the results, since it produced sleep anomalies that occluded those possibly caused by the neurotoxin (Garcia-Garcia et al., 2005); but see (Yi et al., 2007). For this reason, particular care should be taken to include appropriate controls when employing the rotenone model.

In the rat, consumption of seeds from the plant *Cycas micronesica* (cycad) results in degeneration and accumulation of α-synuclein aggregates in dopaminergic and noradrenergic neurons of the SNc and LC (Shen et al., 2010). Sleep analysis performed in this model reported increased NREM and REM sleep during the active 12-h phase. These changes were associated with increased average duration of NREM sleep and increased number of REM episodes (McDowell et al., 2010). Notably, the same study also reported a reduction in the number of orexin neurons in the LH of cycad-fed rats (McDowell et al., 2010), a change which is in line with previous work in orexin depleted rats (Gerashchenko et al., 2001) and may contribute to the observed sleep abnormalities.

Gerashchenko et al. utilized the hypocretin2-saporin neurotoxin (see *Sleep Disturbances in the 6-Hydroxydopamine Model of Parkinson’s Disease*) (Gerashchenko et al., 2001) to lesion substantia nigra and VTA, thereby eliminating a large proportion of neurons (dopaminergic and non-dopaminergic) in both regions (Gerashchenko et al., 2006). In the substantia nigra, but not in the VTA, this intervention led to increased wakefulness and reduced amounts of NREM and REM sleep during light-on and light-off periods. These results are indicative of insomnia and are in part consistent with findings in rats with a selective dopamine lesion of SNc (see *Sleep Disturbances in the 6-Hydroxydopamine Model of Parkinson’s Disease* and *Sleep Disturbances in the 1-Methyl-4-Phenyl-1,2,3,6-Tetrahydropyridine Model of Parkinson’s Disease*) (Lima et al., 2007; Ciric et al., 2019).

#### Genetic Models of Parkinson’s Disease

Genetic models are commonly obtained by overexpressing genes linked to PD, or mutations implicated in autosomal-dominant or recessive forms of the disease. Additional approaches include interventions disrupting mitochondrial function or specific transcription factors (Dawson et al., 2010; Chesselet and Richter, 2011). These strategies allow the study of effects produced by molecular modifications implicated in familial forms of PD, and to reproduce the accumulation of α-synuclein, which is a hallmark of PD absent in 6-OHDA- or acute MPTP-based models (Dawson et al., 2010; Tieu, 2011). The neuronal loss observed in genetic models of PD is generally less pronounced in comparison to that achieved with neurotoxins, particularly 6-OHDA (Dawson et al., 2010; Chesselet and Richter, 2011). However, this does not necessarily represent a limitation to their use, since PD related sleep disorders are often manifested during the early phase of the disease.

In the mouse, overexpression of human α-synuclein driven by the Thy-1 promoter (Thy1-αSyn) leads to a progressive disruption of the nigrostriatal pathway, resulting in a 40% decrease of striatal dopamine (Chesselet et al., 2012). McDowell et al. examined sleep structure in Thy1-αSyn mice and found reduced REM sleep during the 24-h sleep-wake cycle, accompanied by increased NREM sleep during the light-on, inactive period (McDowell et al., 2014). The same authors also observed increased activity during the light-off, wake phase (McDowell et al., 2014). However, analysis of circadian rest-activity performed in Thy1-αSyn mice reported reduced wheel-running particularly pronounced during dark (Kudo et al., 2011). This discrepancy may be accounted for by the different parameters employed to assess the active state of the animals, i.e., whole-body movements detected by telemetry (McDowell et al., 2014) *vs*. wheel-running (Kudo et al., 2011). Notably, both EEG (McDowell et al., 2014) and visual (Kudo et al., 2011) analysis of Thy1-αSyn mice revealed a delay in sleep onset, although during different phases of the 24-h light-dark period.

Circadian rhythm has been also examined in the MitoPark mouse model of PD, in which dopamine neurons are progressively eliminated by selective impairment of mitochondrial function (Ekstrand and Galter, 2009). MitoPark mice showed a general reduction of locomotor activity in concomitance with the gradual loss of dopamine neurons, accompanied by a severe disruption of circadian rhythm in constant darkness (Fifel and Cooper, 2014). A similar loss of endogenous rhythmicity has been observed in neurotoxin models of PD based on striatal (Masini et al., 2017) or ventricular (Gravotta et al., 2011) injection of 6-OHDA. Further studies will be necessary to understand the mechanisms at the basis of these abnormalities as well as their impact on sleep function and architecture.

Mice with reduced (95%) levels of the vesicular monoamine transporter 2 (VMAT2) undergo nigrostriatal dysfunctions affecting the dopaminergic system, which replicate key pathologic features of PD (Caudle et al., 2007). This model displays a number of non-motor symptoms associated with PD, including anomalies of circadian and sleep function (Taylor et al., 2009). VMAT2-depleted mice show reduced ambulation during the active phase of the circadian period (Taylor et al., 2009). They also show a reduced latency to behavioral signs of sleep (Taylor et al., 2009), which contrasts with the delayed sleep onset observed in Thy1-αSyn mice (Kudo et al., 2011; McDowell et al., 2014). Notably, VMAT2-deficient mice show a concomitant reduction of dopamine, noradrenaline, and serotonin in striatum, hippocampus, and cortex (Taylor et al., 2009), which collectively may contribute to the wide range of non-motor symptoms observed in this model.

Altogether, the different findings reported in genetic models of PD fail to provide a coherent representation of one or more specific sleep alterations associated with PD. Aside from the contrasting effects on activity state observed in Thy1-αSyn mice, which may be attributable to different methodological approaches (see above), these inconsistencies are possibly a consequence of distinct genetic strategies (overexpression of α-synuclein as opposed to downregulation of VAMT2), which may produce unique alterations besides those affecting the dopaminergic system.

## Conclusions and Perspectives

Rodent models represent a simple and versatile tool to study not only the classic motor symptoms and complications (i.e., dyskinesia) of PD, but also a number of non-motor co-morbidities. Nonetheless, the development of rat and mouse models of PD-related sleep disturbances has lagged behind in comparison, for instance, to affective and cognitive symptoms. The reason for this lies in part in the complexity of sleep-related disorders, both with regard to their behavioral manifestation and their relationship to distinct cellular and anatomical substrates. The difference in sleep architecture between rodents and humans represents an additional obstacle when interpreting results. In this regard, modeling PD in non-human primates constitutes a clear advantage, since in these animals, sleep structure and EEG patterns closely reproduce the consolidated, monophasic organization observed in humans (Hsieh et al., 2008), which contrasts with the more dispersed distribution of sleep states in rodents (Fifel et al., 2016).

A critical issue related to rat and mouse models, and more in general to all current animal models, is the difficulty to recapitulate the diversified nature of the degenerative processes at the basis of PD, thereby offering the possibility of examining the concomitant involvement of multiple neurotransmitter systems in sleep alterations. Thus, it is not surprising that rodent models of PD do not reproduce the full spectrum of sleep abnormalities associated with PD. However, in spite of these limitations some of these ailments have been reproduced in rats (**Table 1**). For instance, decreased SWS during the quiescent period, suggestive of insomnia, has been described after 6-OHDA-lesion of the MFB (Vo et al., 2014) and in the rotenone model (Yi et al., 2007). Moreover, 6-OHDA-lesion of the VTA (Sakata et al., 2002), as well as administration of rotenone (Yi et al., 2007), or cycad toxin (Shen et al., 2010) increase NREM and REM sleep during the active phase of the circadian cycle, which is indicative of EDS. These results are in line with the analysis of rest-wake circadian rhythm in mice with a bilateral striatal 6-OHDA-lesion, which show decreased activity during the active 12-h period (Masini et al., 2017).

**Table 1:** Sleep disturbances in neurotoxin and genetic rodent models of Parkinson’s disease.

Model	Procedure	Pathology	Wake	NREM/SWS sleep	REM sleep	Species	Reference
**6-OHDA**	Injection (200 µg in 20 µl) in the lateral ventricle	Decreased DA levels in DS (≈40%) ventral striatum (≈50%)	Decreased in light-on	Increased in light-on	Unaltered	Rat	Monti et al., 1999
**6-OHDA**	Bilateral injection in VTA (with desipramine)	Decreased DA neurons in VTA	Decreased in light-off	Increased in light-off	Increased in light-off and decreased in light-on	Rat	Sakata et al., 2002
**6-OHDA**	Unilateral injection in MFB (with desipramine)	Decreased DA neurons in SNc (unilateral, ≥95%)	Increased total time (24 h)	Decrease in light-on	Increased muscle activity	Rat	Vo et al., 2014
**6-OHDA**	Bilateral injections (12 µg/0.5 µl) in DS	Decreased (≈70%) DA neurons in SNc, reduced (27 to 39%) orexin neurons in LH	Unaltered	Unaltered duration (↓breathing frequency during sleep)	Unaltered duration (↓breathing frequency during sleep)	Rat	Oliveira et al., 2018
**6-OHDA**	Partial unilateral lesion of DS—sleep states identified by breathing rat fluctuations	Decreased levels of DA in the DS (unilateral, 51%)	NI	Duration unaltered	Duration unaltered	Rat	Zhurakovskaya et al., 2019
**6-OHDA**	Unilateral injection in SNc (12 or 24 µg)	Decreased DA neurons (56 to >92%) along the SNc rostro-caudal axis (unilateral)	Increased in light-on (↑bout number)	Unaltered	Unaltered	Rat	Ciric et al., 2019
**MPTP**	Injection (200 µg/2 µl) in the SNc	Transient (24 h) decrease (50%) of DA neurons in the SNc (unilateral)	NI	Decrease in latency of light-on and off (up to 5 days after lesion)	Transient (2 days) increase of latency in light-on and off	Rat	Lima et al., 2007
**MPTP**	Five daily systemic (i.p.) injections (25 mg/kg)—test 20 days after toxin treatment	Decreased DA neurons in SNc (30%)	Unchanged (↓bout number but ↑bout length)	Unchanged	Increased in light-on and off (↑bout length)	Mouse	Monaca et al., 2004
**MPTP**	Five daily systemic (i.p.) injections (25 mg/kg)—test 20 and 40 days after toxin treatment	Decreased DA neurons in SNc (30%) and terminals in DS (50%) up to 60 days after toxin treatment	Unaltered	Unaltered	Increased in dark (↑bout number) at day 20, but not at day 40, after toxin treatment	Mouse	Laloux et al., 2008
**MPTP**	One systemic (s.c.) injection (40 mg/kg)	Decreased DA neurons in SNc (78%) and VTA (54%) after 17 days	Increased in light-off at day 14 after toxin treatment	Decreased in light-off at day 14 after toxin treatment	No change	Mouse	Revishchin et al., 2016
**Rotenone**	Infusion (s.c.) 3 mg/kg/day for 28 days	Decrease (≈70%) of DA neurons in SNc	Decreased in dark and increased in light	Increased in dark (↑bout duration) and decreased in light (↓bout number)	Increased in dark (↑bout number)	Rat	Yi et al., 2007
**Cycad**	Feeding with pellet (1.25 g) for 22 weeks	α-syn aggregates in DA neurons, decreased DA neurons in the SNc and DA fibers in DS, reduced orexin neurons in the LH	Decreased in light-off (35% ↑bout number and 50% ↓bout duration)	Increased in light-off (↑bout duration)	Increased in light-off (↑bout number)	Rat	McDowell et al., 2010
**Hypocretin2-saporin**	Injection in SN and VTA	Non-selective neuronal depletion in both regions	Increased during light-on and off (only in SN-lesion)	Decreased during light-on and off (only in SN-lesion)	Decreased during light-on and off (only in SN-lesion)	Rat	Gerashchenko et al. (2006)
**Thy-1 α-Syn**	Transgenic	α-syn aggregates in SNc and 40% reduction of striatal DA at 14 months	Increased active wake in light-off	Increased in light (↑bout length)—↑bout length in dark	Decreased in light-off (↓bout number) and light-on	Mouse	McDowell et al., 2014
**VMAT2-deficiency**	Transgenic	Decreased DA, NE, and 5-HT in DS, hippocampus, and cortex at 12–15 months	Decreased at 4–6 months	Decreased sleep latency		Mouse	Taylor et al., 2009

NI, not investigated; i.p., intraperitoneal; s.c., subcutaneous; SN, substantia nigra; DA, dopamine; NE, noradrenaline; 5-HT, serotonin; α-syn, α-synuclein; *↑*, increased; *↓*, decreased. See text for other abbreviations.

Modifications in sleep architecture have also been observed in mice and rats intoxicated with MPTP (Monaca et al., 2004; Lima et al., 2007; Laloux et al., 2008; Revishchin et al., 2016) (**Table 1**). However, MPTP-based approaches have failed to provide consistent results and are often observed only during a limited period of time following administration of the toxin (Lima et al., 2007; Laloux et al., 2008). This limited efficacy is in line with the lack of alterations of circadian locomotor activity rhythm reported in mice following acute or chronic treatment with MPTP (Fifel et al., 2013).

How can rodent modeling of sleep disorders in PD be improved? Overall, a general effort should be made to refine and harmonize the different approaches used to generate neurotoxin-based models. In the case of 6-OHDA, for instance, rats have been treated with i.c.v. injection, unilateral and bilateral injections in MFB, distinct midbrain dopaminergic nuclei (i.e., SNc and VTA), or in the striatum (see **Table 1**). Moreover, some studies (Sakata et al., 2002; Vo et al., 2014) have used pretreatment with desipramine, a selective inhibitor of noradrenaline reuptake. The different degree of dopamine and noradrenaline depletion produced by these interventions may cause distinct effects in sleep behavior, thereby contributing to discrepancies and confounding interpretations.

Combined loss of dopamine, noradrenaline, and to some extent functional inactivation of serotonin has been achieved with neurotoxins (Breese and Traylor, 1971; Reader and Gauthier, 1984; Santiago et al., 2010; Bonito-Oliva et al., 2013). In addition, 6-OHDA has been found to reduce the number of orexin/hypocretin neurons in the LH (Cui et al., 2010; Oliveira et al., 2018). Therefore, some of the sleep abnormalities observed in rodent models of PD may depend on the loss, or impairment, of one or more of these components of the arousal system. However, several key structures implicated in sleep regulation, including cholinergic and histaminergic nuclei, are spared by neurotoxin or genetic interventions. Therefore it will be important to strengthen construct validity of rodent models by establishing a more stringent link between the observed sleep abnormalities and PD-related biological dysfunctions.

The limitation posed by the relatively restricted neurodegenerative effects observed in most experimental models has been in part circumvented by acting on specific targets. For instance, bilateral injection of ibotenic acid in the PPN has been proposed to represent a model of PD cholinopathy (Ciric et al., 2018). In the rat, this intervention results in altered microstructure of both NREM and REM sleep during the inactive phase (Ciric et al., 2018). However, the neurotoxic effect exerted by ibotenic acid in the PPN is likely to affect not only cholinergic neurons, but also glutamatergic and GABAergic cells.

Chemo- and optogenetics techniques offer a particularly attractive approach to parse the involvement of neuronal networks in PD-related sleep-wake disturbances. For instance, using selective expression of designer receptor exclusively activated by designer drugs (DREADD) (Alexander et al., 2009), Kroeger et al. showed that distinct neuronal populations in the PPN regulate different aspects of sleep-wake behavior (Kroeger et al., 2017). Similar approaches, including optogenetic manipulation, showed that the dopamine neurons of the VTA are required for the initiation and maintenance of wakefulness (Eban-Rothschild et al., 2016; Oishi et al., 2017). Notably, these findings are in line with results obtained in rats with a selective 6-OHDA lesion of the VTA, which display decreased wakefulness during the active, light-off phase (Sakata et al., 2002).

Opto- and chemogenetic interrogation has also been employed to investigate the role in sleep of a population of dopamine neurons located in the dorsal raphe. These cells degenerate in PD (Halliday et al., 1990), but their involvement in non-motor symptoms remains to be in large part assessed. Cho et al. showed that ontogenetic stimulation of these dopamine population induces and maintains wakefulness and that vice versa their chemogenetic inhibition counteracts wakefulness and increases NREM sleep (Cho et al., 2017). In another study, Qiu et al. clarified the contribution to sleep of the external globus pallidus, whose activity is reduced in PD. It was found that, in the rat, activation of this structure resulted in enhanced sleep, possibly mediated *via* inhibition of cortical regions involved in the regulation of arousal nuclei (Qiu et al., 2016).

Recent evidence provides an attractive approach to reproduce the path of PD progression described by Braak et al. (2003). Injection of α-synuclein pathological fibrils in the gut muscle layer, leads to the spread of Lewy bodies from the peripheral nervous system, through the vagus nerve, to the brain, followed by loss of dopamine neurons (Kim et al., 2019). These effects are accompanied by motor impairment and by non-motor symptoms, including cognitive and affective deficits (Kim et al., 2019). The widespread distribution of α-synuclein fibrils, which are observed in several structures including the LC, suggests the potential use in the future of this or similar models for the study of sleep-related co-morbidities in PD.

One particularly interesting aspect of sleep disorders in PD is their potential impact on other frequent non-motor symptoms, such as cognitive and affective disorders. Indeed, cycling and coordinated transition of NREM and REM sleep are implicated in learning and memory processes (Moroni et al., 2014; Boyce et al., 2016). The synchronous activation of hippocampal pyramidal neurons during sleep results in the propagation of information throughout the neocortex, thereby leading to memory consolidation (Buzsaki, 1989). Depression has also been correlated to reduced SWS and increased duration and intensity of REM sleep (Riemann et al., 2001). The existence of a causative link between PD-related sleep disruptions and psychiatric co-morbidities remain to be further investigated. In this regard, it should be mentioned that memory deficit, depression, and anxiety are commonly observed in rodent models of PD, which may therefore represent valuable tools to help clarifying some of these questions.

## Author Contributions

GF conceived the presented idea and supervised the project. DM and GF wrote the manuscript. CA and MM provided critical feedback and helped shape the manuscript. All authors approved the final version for submission.

## Funding

This work was supported by the Swedish Research Council (grant 2015-02886), the Swedish Brain Foundation and the Swedish Parkinson Foundation (GF). This work is part of a joint Brazilian-Swedish Research Collaboration supported by the Swedish Foundation for International Cooperation in Research and Higher Education (STINT) and by the Coordenação de Aperfeiçoamento de Pessoal de Nível Superior (CAPES), Brazil.

## Conflict of Interest

The authors declare that the submitted work was carried out in the absence of any personal, professional or financial relationships that could potentially be construed as a conflict of interest.
